# Potential Application of Cornelian Cherry Extract on Broiler Chickens: Growth, Expression of Antioxidant Biomarker and Glucose Transport Genes, and Oxidative Stability of Frozen Meat

**DOI:** 10.3390/ani11041038

**Published:** 2021-04-07

**Authors:** Doaa Ibrahim, Amira Moustafa, Aya Sh. Metwally, Mohamed A. Nassan, Karima Abdallah, Fatma Eldemery, Vincenzo Tufarelli, Vito Laudadio, Asmaa T. Y. Kishawy

**Affiliations:** 1 Department of Nutrition and Clinical Nutrition, Faculty of Veterinary Medicine, Zagazig University, Zagazig 44519, Egypt; 2 Department of Physiology, Faculty of Veterinary Medicine, Zagazig University, Zagazig 44519, Egypt; amiramostafa@zu.edu.eg; 3 Department of Pharmacology, Faculty of Veterinary Medicine, Aswan University, Aswan 81511, Egypt; ayashawky@aswu.edu.eg; 4 Department of Clinical Laboratory Sciences, Turabah University College, Taif University, P.O. Box 11099, Taif 21944, Saudi Arabia; m.nassan@tu.edu.sa; 5 Department of Food Control, Faculty of Veterinary Medicine, Zagazig University, Zagazig 44519, Egypt; karimaeissa1989@yahoo.com; 6 Department of Hygiene and Zoonoses, Faculty of Veterinary Medicine, Mansoura University, Mansoura 35516, Egypt; Fatmaelbaz@mans.edu.eg; 7 Department of DETO, Section of Veterinary Science and Animal Production, University of Bari, Strada Provinciale per Casamassima km 3, 70010 Valenzano, Italy; vincenzo.tufarelli@uniba.it (V.T.); vito.laudadio@uniba.it (V.L.)

**Keywords:** cornelian cherry extract, glucose transporter, gene expression, oxidative biomarker, chicken

## Abstract

**Simple Summary:**

Supplementation of the poultry diet with plant extracts rich in polyphenolic compounds could improve the performance of animals as well as the oxidative stability of their derived meat. The present study evaluated the efficacy of cornelian cherry extract (CCE) on the expression of genes controlling glucose transporters and different assays regulating the oxidative stability of frozen, stored meat over a long period of time (90 days of storage). The results indicated that the addition of 200 mg/kg of CCE to the diet could improve the growth rate and antioxidant status of broiler chickens and thus increase their productivity. Moreover, polyphenolic compounds rich in CCE can act as antioxidant agents to increase the shelf-life extension of frozen, stored poultry meat. Finally, supplementation with CCE could increase the total concentration of phenolic compounds in poultry meat offered to human consumers.

**Abstract:**

The use of natural plant extracts in poultry feed could improve their productivity as well as the oxidative stability of stored derived meat. The roles of cornelian cherry extract (CCE) in growth, cecal microbes, and meat antioxidative markers of broiler chickens were evaluated. A total of 500 Ross 308 broiler chicks were fed diets supplemented with CCE (0, 50, 100, 200, 400 mg/kg of diet) for 38 days. The highest levels of weight gain and feed utilization were observed in a group fed 200 mg/kg of CCE. Maximum upregulation of glucose transporters—1 and 2 and sodium-dependent glucose transporter genes—were found in the group fed 200 mg/kg of CCE. *Lactobacilli* and *Bifidobacterium* colonization increased as the CCE levels increased. The greatest upregulation of antioxidant genes (glutathione peroxidase, catalase, and superoxide dismutase) in breast meat was observed in groups fed CCE (200 and 400 mg/kg). Dietary CCE significantly delayed the lipid oxidation of breast meat compared with that of the control group. The total phenolic content, 2,2-Diphenyl-1-Picrihydrzyl (DPPH) radical scavenging activity and reducing power in meat improved with higher levels of CCE. Dietary CCE improved the growth, performance of broilers, and meat antioxidant stability after 90 days of storage.

## 1. Introduction

The intention for the widespread use of phytonutrients in the poultry industry is ultimately associated with the growing discouragement of the use of antibiotics in feed. Recently, natural, active, plant-derived compounds have been gaining great importance, because of their ability to enhance poultry growth performance by improving nutrient digestibility, increasing the concentration of nutrient transporters, sustaining a healthy gut environment, and improving the quality of their products [1]. A phytogenic diet has also been reported to produce changes in the cell membrane permeability, resulting in a higher absorption rate of micronutrients from the small intestine [2]. Additionally, herbal and medicinal plant additives might have the capacity to control intestinal pathogenic bacteria and improve the beneficial intestinal microbiota [3,4,5] due to their antimicrobial, fungicidal, antiviral, anticoccidial, and antioxidant properties [6,7]. Furthermore, plant extracts that are rich in polyphenols can be effective for preserving meat and their products against oxidative deterioration, pathogen growth, and bacterial spoilage [8].

On the other hand, modern large-scale broiler production prompts stressful conditions such as high-stocking density, heat stress, immunological challenges, handling, feed quality, and transportation [9]. These stressors can enhance reactive oxygen species (ROS) production and interrupt the balance between the antioxidant defense systems and oxidation inside a bird’s body, causing oxidative stress [10]. The harmful effect of oxidative stress can be reduced by the dietary inclusion of antioxidants [5]. The use of natural plant-derived compounds rich in polyphenolic compounds can improve the antioxidative status of the living birds and increase the oxidative stability of their derived meat [11].

Among these natural compounds is cornelian cherry (*Cornus mas*) extract (CCE), which is composed of several active compounds, including five anthocyanins: delphinidin 3-galactoside, cyanidin 3-rhamnosylgalactoside, cyanidin 3-galactoside, pelargonidin 3-rhamnosylgalactoside, and pelargonidin 3-galactoside [12]. Ursolic acid is an important constituent of CCE [13,14,15,16] possessing antioxidant and antibacterial properties. Additionally, it contains vitamin C, trace minerals, organic acids, pectintriterpenoids, iridoids, pectins, and tannins that range within the safe standards levels of food [17,18,19]. It is rich in flavonoids such as quercetin 3-O-rhamnoside, quercetin 3-O-rutinoside, and quercetin 3-O-glucuronide [20] and phenolic compounds such as caffeic acid, caffeoylquinic acids, p-coumaric acid, and ellagic acid [21]. Furthermore, CCE has been shown to have antimicrobial [22] anti-inflammatory, and antioxidant activity [23] as well as a hypoglycemic effect [24]. Moreover, the high content of iridoids, such as cyclopentanopyran, found in CCE provides pharmacological properties such as antibiotic and anti-inflammatory effects [25].

Poultry meat is highly susceptible to quality deterioration by lipid oxidation during storage, leading to a decrease in nutritive value and the production of a high content of lipid oxidation products [26]. The oxidative stability of poultry meat is influenced by birds’ diets and dietary inclusion of CCE with an abundant amount of polyphenolic bioactive compounds that have been demonstrated to scavenge free radicals and chelate metal ions, helping to increase the oxidation resistance of meat. Moreover, the application of CCE to broiler breast meat was associated with a lower thiobarbituric acid reactive substances (TBARS) value and an increased meat shelf life [15].

Digested carbohydrates, protein, and lipids are transferred into the body by certain transporter proteins located in the brush border enterocytes of the small intestine [27,28]. These include GLUT1 and GLUT2, which are responsible for monosaccharide transportation (glucose, galactose, fructose, and mannose) across the intestinal membrane [29]. The greater expression of transporter-encoding genes leads to a higher flood of nutrients into the intestinal cells and, subsequently, into the blood [30].

Cornelian cherry extract can play an important role in chickens’ intestinal health and meat quality owing to its active principle content. Thus, this work investigated different mechanisms and provides new data about the effects of cornelian cherry extract on growth performance, glucose transporters, gut microbes, and meat oxidative stability in broiler chickens.

## 2. Materials and Methods

The management practices and procedures followed animal welfare, ethical norms, and guidelines of the Institutional Animal Care and Use Committee of the Faculty of Veterinary Medicine at Zagazig University.

### 2.1. Birds, Diets, and Experimental Design

A total of 500 male Ross broiler chicks (ROSS 308), on the day of hatching (initial body weight 45.8 ± 1), were purchased from a commercial hatchery. Chicks were weighed and randomly divided into five treatment groups with 10 replicate pens containing 10 birds each. The study was organized at the Faculty of Veterinary Medicine, Zagazig University, Egypt. The experimental protocol was accepted by the ethics committee of the Institutional Animal Care and Use Committee of Zagazig University, Egypt. All animal experiments were done according to the recommendations described in “The Guide for the Care and Use of Laboratory Animals in scientific investigations” to ensure their welfare, maintain their rights, and cause minimal stress. All chicks were housed in the same environmental and sanitary conditions all over the experimental period. Birds were raised in floor pens with wood shavings (bird density: 10 broilers/m^2^) in an environmentally controlled room. The photoperiod in all experimental pens was maintained at 23 L:1 D h for the first 3 days, followed by 20 L:4 D h until the end of the experiment. The relative humidity ranged from 65 to 75% throughout the trial. During the 1st week, the room temperature was initially set at 33 °C and then gradually decreased until the final temperature of 23 °C was reached. The control starter (d 1–10 d) and grower-finisher (d 11–38) diets were formulated to cover the nutrient requirements of Ross broilers according to the nutritional specifications of ROSS [31]. All birds were allowed access to water and feed ad libitum. The birds were offered a basal diet supplemented with (0 (control), 50, 100, 200, and 400 mg/kg diet of cornelian cherry extract (CCE). The quantities of feed ingredients and the chemical composition of the control diet are listed in Table 1. The proximate analysis of the feed ingredients was done according to the standard procedures of the Association of Official Agricultural Chemists [32]. Asiatic cornelian cherry extract was obtained from Shaanxi Sinuote Biotech Co. Ltd. China. The extract was collected by water alcohol extraction with an extraction ratio of 10:1. The HPLC analysis of the extract based on the manufacturing company was 100 g of cornelian cherry extract containing 203 parts per thousand (PPT) iridoids (consisting of 85% loganic acid), 2.8 PPT ellagic acid, 8.9 PPT anthocyanins, and 4.1 PPT flavonols such as quercetin 3-glucuronide, kaempferol galactoside, and kaempferol glucoside.

### 2.2. Growth Parameters and Digestibility Trial

The body weight and feed intake (FI) were estimated during the starter period (d 1–10) and grower–finisher period (d 11–38) to calculate the body weight gain, FI, and feed conversion ratio for the whole experimental period (d 1–38). The apparent nutrient digestibility was determined with titanium oxide. At 38 days of age, titanium oxide was added to experimental diets at a rate of 5 g/kg diet. The excreta from each replicate pen was collected every 8 h for five days and analyzed for dry matter, crude protein, ether extract, and crude fiber according to the Association of Official Agricultural Chemists [32]. The titanium oxide content in the diets and excreta was analyzed spectrophotometrically after acid digestion in accordance with the method presented by Short et al. [33] The apparent digestibility coefficient of nutrients was calculated in accordance with the equation presented by McDonald [34].

Apparent nutrient digestibility = 100 − [100 × (Indicator content (diet)/Indicator content (feces) × Nutrient content (feces)/Nutrient content (diet)](1)

### 2.3. Sample Collection and Analytical Procedures

At the end of the experimental period, randomly selected birds were weighed and slaughtered.

For serum biochemical measurements, 3 mL blood samples were collected from each bird and then centrifuged for 15 min at 2000 rpm. Clear serum samples were kept at −20 °C until further biochemical analysis.

For meat chemical composition analysis, meat samples were collected from the breast and thigh and then stored at −20 °C until chemical analysis.

For the meat antioxidant analysis, breast meat samples were frozen immediately until the total phenolic content (TPC) and thiobarbituric acid reactive substance (TBARS) content were analyzed and the 2,2-Diphenyl-1-picrihydrzyl (DPPH) assay and Ferric reducing antioxidant power (FRAP) were conducted at 7 and 90 days of storage at −20 °C.

For the molecular analysis, breast meat samples were collected and stored at −80 °C until the analysis of antioxidant-related genes. The small intestine (jejunal part) was separated and the digesta was squeezed out from it and rinsed 3 times in PBS (NaH_2_PO_4_, 1.47 mmol/L; Na_2_HPO_4_, 8.09 mmol/L; and NaCl, 145 mmol/L). One square centimeter of the distal jejunum immediately before the Meckel’s diverticulum was dissected and kept in Trizol reagent at −80 °C until the analysis of nutrient transporter encoding genes.

### 2.4. Serum Biochemical Analysis

Serum aspartate aminotransferase (AST), and alanine aminotransferase (ALT), total cholesterol (TC), triglycerides (TGs), high-density lipoprotein cholesterol (HDL-C), low-density lipoprotein cholesterol (LDL-C), very-low-density lipoprotein cholesterol (VLDL-C), creatinine, uric acid, total protein, globulin, and albumin concentrations were determined using commercial diagnostic kits (Spinreact Co., Santa Coloma, Spain).

### 2.5. Chemical Copmostion of Meat

The dry matter, crude protein, fat and ash contents of breast and thigh meat were analyzed according to the Association of Official Analytical Chemists (AOAC) [32].

### 2.6. Antioxidant Potential of Broiler Meat

Breast meat samples (5 g) were mixed with phosphate buffer (20 mL; pH 7.4) and glycerol (20 mL; 20%) and homogenized and filtered to ensure they were free from connective tissues.

#### 2.6.1. Total Phenolic Content (TPC)

The total phenolic content of breast meat was measured in accordance with [35]. Briefly, a previously prepared meat sample (100 μL), distilled water, 2.5 mL of 95% ethanol (500 μL), and 50% Folin–Ciocalteu reagent (250 μL) were mixed. This mixture settled for 5 min, and then 5% Na_2_CO_3_ (500 μL) was added. The mixture was rotated in a vortex meter and left in a dark place for 1 h. The absorbance of samples was measured at 725 nm via a spectrophotometer. The quantity of TPC in meat was measured as Gallic acid equivalents (milligrams of gallic acid per 100 g of meat sample).

#### 2.6.2. 2,2-Diphenyl-1-picrihydrzyl (DPPH) Assay

The DPPH scavenging capacity of the meat sample was calculated as described by [36]. The DPPH A solution (0.25 mM) was formed in methanol. Each sample (100 mL) was mixed with 100 μL of DPPH solution and maintained for 30 min at 25 °C in a dark place, and the sample absorbance was read at 517 nm by a spectrophotometer. The scavenging activity percentage of DPPH in the meat was determined with the following equation:

Scavenging activity of DPPH (%) = 1 − [A_1_ − A_2_] × 100 (2)

Blank absorbance

A_1_ = Sample absorbance; A_2_ = Blank absorbance

#### 2.6.3. Ferric Reducing Antioxidant Power (FRAP)

FRAP in meat was detected according to Oyaizu [37]. A 200-μL homogenized meat sample was mixed with sodium phosphate buffer (500 μL). After that, the prepared solution was maintained in a water bath for 20 min at 50 °C and then centrifuged for 10 min with trichloroacetic acid (2.5 mL) and ferric chloride solution (100 μL). The spectrophotometric absorbance of the samples was measured at 700 nm. The FRAP was estimated as μmol/Fe^2^+/g meat.

### 2.7. TBARS Assay

Lipid oxidation in breast meat was evaluated based on the malondialdehyde (MDA) content, as the MDA concentration in breast meat was determined as previously described by [38]. Briefly, perchloric acid (27 mL) was added to 5 g breast meat samples and then homogenized and filtered. Supernatant samples were mixed with thiobarbituric acid (2 mL) and incubated for 20 min in a water bath (100 °C). Consequently, direct cooling and centrifugation were done for 15 min, and the absorbance was measured by a spectrophotometer at 532 nm. The values are expressed as milligrams of malondialdehyde per kilogram of meat.

### 2.8. Real-Time PCR to Assess Nutrient Transporter Encoding Genes

Total mRNA was extracted from jejunum and breast meat samples (*n* = 10 per treatment) using Trizol reagent (TaKaRa Biotechnology Co. Ltd., Dalian, Liaoning, China). The isolated RNA was treated with the RNeasy Mini Kit; Qiagen, Cat. No. 74104 according to the manufacturer’s guidelines. The quantity and purity of the total RNA were determined by a NanoDrop ND-8000 spectrophotometer (Thermo Fisher Scientific, Waltham, USA). Complementary DNA (cDNA) was obtained by reverse-transcription of isolated RNA samples using RevertAidTM H Minus kits (Fermentas Life Science, Pittsburgh, PA, USA). One microliter of this cDNA was mixed with 2× maxima SYBR Green PCR mix (12.5 μL) and RNase free water (10.5 μL), and then, 0.5 μL of each forward and reverse primer for the selected genes was added. The primers’ sequences of genes encoding the glucose transporter and antioxidant enzymes are described in Table 2. Glyceraldehyde-3-phosphate dehydrogenase (GAPDH) was selected as a reference gene.

### 2.9. Microbiological Assay

Spread plate counting method for different microbes: One gram of cecal content was mixed with 9 mL phosphate-buffered saline and vortexed for 1 min. Samples were serially diluted in sterile diluents (0.5 g/kg peptone water in distilled water). De Man, Rogosa, and Sharpe (MRS, CM1153, Oxoid, Basingstoke Hampshire, UK) agar medium was used for the enumeration of *Lactobacilli* (MRS, CM1153, Oxoid), Bifidus selective agar was utilized to determine the *Bifidobacteria* content (BSM-Agar, 88517, Sigma, St. Louis, MO, USA), and violet-red bile glucose agar (VRBG, CM485, Oxoid) was used to determine the *Escherichia coli* content. After incubation under appropriate conditions for each group of bacteria (72 h at 37 °C under anaerobic conditions for *lactobacilli* and *Bifidobacteria* and 48 h at 39 °C under aerobic conditions for *E*. *coli*), colonies were counted on the plates, and the microbial population was expressed as log_10_ CFU/g of cecal content.

### 2.10. Statistical Analysis

The data analysis was conducted using the general linear model (GLM) procedure of Statistical Package for the Social Sciences, software (SPSS), after confirming the homogeneity among experimental groups using the Levene test and the normality using the Shapiro–Wilk test. Tukey’s test was used to test for significant differences between the mean values. All results are expressed as the standard error of the mean (SEM), and the statistical significance was set at *p* < 0.05. Cecal colony-forming unit (CFU) data were converted to log_10_ CFU numbers before analysis. The fold change was measured by the following equation: (B–A)/A where the lowest value is A and the highest value is B. Relative fold changes in the expression of target genes were calculated by the 2^−ΔΔ^Ct method using the GAPDH gene as an internal control gene to normalize the target gene expression levels [39].

## 3. Results

### 3.1. Growth Performance and Nutrient Digestibility

The growth performance parameters of the total growing period and nutrient digestibility are shown in Table 3. The body weight gain (BWG) was significantly greater (*p* < 0.05) in groups fed 100, 200, and 400 mg/kg of CCE when compared with the control group. Moreover, the highest BWG was observed in the group fed 200 mg/kg of CCE (increased by 9% in comparison with the control group). The feed conversion ratio (FCR) was improved in all groups fed CCE supplemented diets at different levels, and the biggest improvement in FCR was detected in the group fed diets supplemented with 100 or 200 mg/kg CCE. Concerning the nutrient digestibility, the groups fed 100 or 200 mg/kg CCE showed higher dry matter (DM) contents and CP digestibility levels in comparison with diets containing other levels of CCE, while the control group showed the lowest level of DM digestibility. No significant difference in the digestibility of CF was observed among the different groups.

### 3.2. Serum Biochemical Parameters

Data regarding the impact of CCE on serum biochemical parameters after 38 days are shown in Table 4. The levels of serum AST, ALT, creatinine, uric acid, total protein, albumin, globulin, TGs, HDL-C, and VLDL-C were not affected by dietary CCE (*p* > 0.05). However, total cholesterol and LDL-C concentrations were significantly reduced (*p* < 0.05) in the group supplemented with 400 mg/kg of CCE in comparison with the other groups.

### 3.3. Chemical Composition of Meat:

The dry matter, crude protein, fat, and ash contents both breast and thigh meat were not affected (*p* > 0.05) by dietary CCE, as shown in Table 5.

### 3.4. Gene Expression of Glucose Transporter

**Figure 1:**
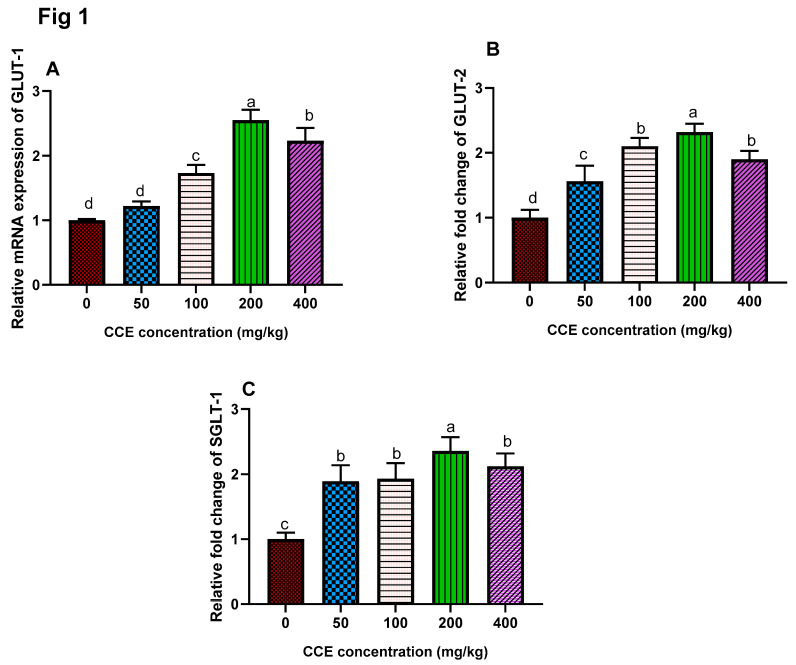
Effect of different levels of cornelian cherry extract (CCE) on glucose transporters genes expression (glucose transporter-1 (**A**) *GLUT-1*, glucose transporter-2 (**B**) *GLUT-2*, Sodium-dependent glucose transporter (**C**) *SGLT-1*. ^a–d^ Means within the same column carrying different superscripts are significantly different at *p* < 0.05. Values are means ± standard error. Number of birds/replicates = 10

The mRNA expression levels of the jejunal nutrient transport genes (*GLUT1, GLUT2*, and SGLT-1) are presented in Figure 1. The expression levels of glucose transporter genes (*GLUT-1*, *GLUT-2*, and *SGLT-1*) were significantly upregulated in groups fed CCE, and the highest level was found in the group supplemented with 200 mg/kg of CCE (*p* < 0.05) with fold changes of 2.55, 2.32, and 2.36, respectively. The aforementioned upregulation was declined in the group supplemented with 400 mg/kg of CCE. Of note, GLUT1 expression in the group supplemented with 50 mg/kg of CCE showed no significant difference as compared with the control group. Moreover, *SGLT-1* expression was significantly upregulated in all groups supplemented with CCE, and the maximum level of upregulation was found in the group supplemented with 200 mg/kg of CCE.

### 3.5. Gut Microbiota

The gut microbiota data are presented in Table 6. The mean cecal populations of beneficial lactobacilli, bifidobacteria significantly increased after dietary supplementation with 100, 200, or 400 mg/kg of CCE when compared with the control group (*p* < 0.05). The population of *E. coli* in cecal samples significantly decreased (*p* < 0.05) as the CCE level increased, and the lowest reduction of *E. coli* was observed with diets supplemented with 200 or 400 mg/kg of CCE.

### 3.6. Antioxidant-Related Genes

**Figure 2:**
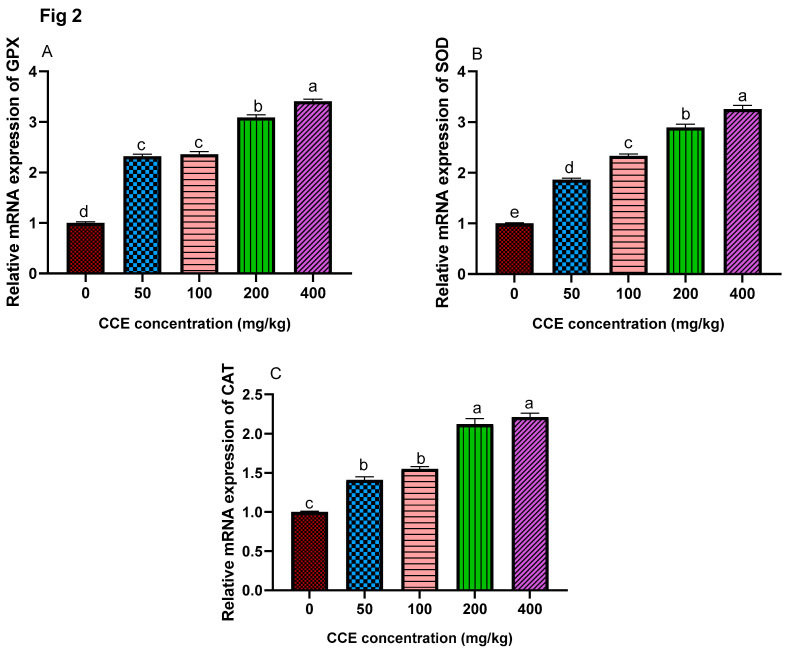
Effect of different levels of cornelian cherry extract (*CCE*) on (glutathione peroxidase, (*GPx*) (**A**), superoxide dismutase, (*SOD*) (**B**) and catalase, (*CAT*) (**C**). ^a–e^ Means with a row carrying different superscripts are significantly different at *p* < 0.05. Values are means ± standard error. Number of birds/replicates = 10.

The expression patterns of selected antioxidant-related genes (glutathione peroxidase, *GPx*, superoxide dismutase, *SOD* and catalase, *CAT*) are presented in Figure 2. The highest expression levels of *GPX* were observed in groups supplemented with 400 mg/kg of CCE followed by the groups fed 200 mg/kg of CCE then 50 and 100 mg/kg of CCE. The mRNA expression of *GPx* was significantly upregulated (*p* < 0.05) in the groups fed 200 or 400 mg/kg of CCE compared with groups fed 50 or 100 mg/kg of CCE when compared with the control group. The mRNA expression level of *SOD* was significantly increased as the CCE level increased when compared with the control. The highest expression levels of catalase were observed in groups supplemented with 200 or 400 mg/kg of CCE followed by the groups fed 50 or 100 mg/kg of CCE when compared with the control group.

### 3.7. Antioxidant Potential of Breast Meat

Data from the analysis of the total phenolic content (TPC), 2,2-diphenyl-1-picrihydrzyl (DPPH) assay, and ferric reducing antioxidant power (FRAP) are presented in Table 5.

#### 3.7.1. Total Phenolic Content (TPC) in Breast Meat

After a short storage period (7 days), the TPC content of breast meat was significantly increased with an increasing level of dietary CCE. After a long storage period (at day 90), the highest values of TPC were observed in the meat of bird groups fed 200 or 400 mg/kg of CCE.

#### 3.7.2. The Free (DPPH) Radical Scavenging Activity

The DPPH activity significantly increased (*p* < 0.05) in breast meat from groups fed dietary CCE in a dose-dependent manner, even after 90 days of storage.

#### 3.7.3. FRAP Reducing Activity

The capacity of the breast myofibrillar protein to reduce Fe^3+^ to Fe^2+^ was greater in the meat of groups fed an increased level of dietary CCE. This capacity was increased after 90 days of storage by 1.1 and 1.7 times, respectively, in the meat from groups fed 200 and 400 mg/kg of CCE when compared to the control group.

### 3.8. Lipid Peroxidation

Lipid peroxidation, determined as the concentration of MDA in breast meat after 7 and 90 days of storage, is presented in Table 7. At day 7 (short term storage), the MDA level was significantly reduced (*p* < 0.05) in all groups supplemented with CCE, and the lowest levels were observed in the groups fed 200 and 400 mg/kg of CCE when compared with the control group. At day 90 (long-term storage), all groups supplemented with CCE, except the group fed 50 mg/kg, showed reduced levels of MDA when compared with the control group. The greatest reduction in MDA was observed in the group fed 400 mg/kg of CCE. Moreover, the meat MDA content in the group supplemented with 400 mg/kg of CCE was decreased by up to 59.5% and 68.6% after 7 and 90 days of storage, respectively, when compared with the control (*p* < 0.05).

## 4. Discussion

Natural plant extracts have been shown to improve the performance of birds by augmenting nutrient utilization and bacterial modulation in the gastrointestinal tract as well as improving the meat quality and oxidative stability. Among these natural products, polyphenols gained growing interest due to their numerous functional properties. Cornelian cherry extract (CCE) has been reported to have an abundance of polyphenolic compounds. The current study demonstrated that CCE could be used to improve the growth rate of broilers by controlling nutrient utilization and absorption and the gut microbiota, and increasing the shelf-life storage of poultry meat by boosting the oxidative stability of meat. In the present study, supplementation of CCE has been shown to improve the feed efficiency of birds by lowering the overall FCR by nearly 5% in the group supplemented with 100 and 200 g/kg CCE. Herein, CCE supplementation has been shown to improve the feed efficiency of birds by lowering the overall FCR by nearly 5% in groups supplemented with 100 or 200 g/kg CCE. The present results are consistent with the results of other researchers [40], who reported that the dietary inclusion of different phenolic compounds for broilers had a positive impact on growth performance parameters. Plant-rich phenolics could promote the growth performance of broilers [40,41] through their potential to improve the antioxidant status of the gut [42]. Furthermore, the application of green tea extract, which is rich in catechins, at concentrations of 100 or 200 mg/kg, in feed has been found to boost the growth performance of broiler chickens [43,44]. Additionally, Herrero-Encinas et al. [45] showed that dietary supplementation with olive extract rich in polyphenolic compounds significantly improved the weight gain and feed conversion ratio of broilers. Similarly, the body weight gain of broiler chickens improved after feeding with grape seed proanthocyanidin extract [46]. Additionally, a sugarcane-served polyphenol mix had a positive effect on the growth performance of broilers and modulated the negative effect of heat exposure [47]. Moreover, the consumption of berries, which have numerous bioactive phenolic compounds, was shown to upregulate the expression of growth-related genes such as insulin-like growth factor binding proteins [48]. Furthermore, certain phytogenic derived agents could improve gastrointestinal barrier function and nutrient absorption [49,50]. Sarker et al. [51] showed that feeding with *Cornus officinalis* had no adverse effect on the growth rate in broilers.

On the other hand, the concentrations of serum AST and ALT indirectly reveal the liver health status and increases in their levels are considered markers of liver damage [52]. In addition, the function of kidney can be estimated via the decrease or increase in of urea and creatinine serum levels. Herein, serum concentration of AST, ALT, uric acid, and creatinine were not affected by dietary CCE and were within normal range, which indicates healthy liver and kidney functions in both control and CCE supplemented groups. Moreover, the current study revealed that higher levels of dietary CCE (400 mg/kg) significantly reduced total cholesterol and LDL-C levels in serum. Similarly, Zhang et al. [53] specified that the feeding of broiler chickens with Chinese bayberry leaves, which are rich in phenolic compounds, significantly reduced the serum cholesterol concentration. The presence of a higher concentration of proanthocyanidins in sorghum, which has antioxidative properties, was also reported to be associated with cholesterol-lowering [54]. Furthermore, the higher concentration of anthocyanins in cornelian cherry powder had a hypercholesterolemic effect in rats via augmenting peroxisome proliferator-activated receptor (PPARα) protein expression and controlling reactive oxygen species (ROS) production and, subsequently, the inflammatory process [55]. Additionally, serum protein and globulin concentrations were not significantly different between treatments and were within the normal range [56]. Similarly, supplementation of broiler chickens with polyphenol extract did not affect their serum protein and globulin concentrations [57]. In addition, supplementation with CCE had no significant effects on the chemical composition (DM, CP, EE and Ash) of meat; these results are in accordance with Gopi et al. [40].

Additionally, the enhanced growth performance of the broilers could have resulted from increasing levels of probiotic bacteria such as *lactobacilli* and *Bifidobacteria*. Moreover, the population of these beneficial bacteria increased in groups supplemented with higher levels of CCE. These positive effects could be related to the role of phenolic-rich cornelian cherry extract on the intestinal microflora, leading to an increase in the concentration of beneficial bacteria (probiotic effect) or inhibiting the growth of pathogenic species (antimicrobial effect) [58]. In agreement with our results, *Bifidobacterium* and *Lactobacillus* have been shown to be the most widely used probiotic bacteria, exerting health-promoting properties, such as the maintenance of gut barrier function [59]. Additionally, an increase in probiotic bacteria, such as *Lactobacillus*, is accompanied by a decrease in the concentration of pathogenic *E. coli*, in accordance with authors [60,61] who stated that *Lactobacillus* can quantitatively inhibit the adherence of pathogenic *E. coli*. The potential effect of phenolic compounds in CCE on the gut microbiota may result from modulation of the bacterial population by acting as prebiotics and enriching the beneficial bacteria [62]. The antimicrobial properties of polyphenols are of primary significance and inhibit biofilm formation in the gut by suppressing harmful bacteria [63]. In vitro testing of the antimicrobial activity of cornelian cherry extract demonstrated its inhibitory effects against *Staphylococcus* aureus and *Escherichia coli* [64]. Caffeic acid, present in CCE, has been described as a potential inhibitor of the growth of *E. coli* and *Clostridium* [65].

Furthermore, flavonoids can change the microbiota ecosystem through their bacteriostatic or bactericidal properties [66]. In addition, blueberry flavonoids can inhibit the activity of *E. coli,* which reduces the integrity of the intestinal barrier as a key mechanism of its pathogenesis [67]. Moreover, feeding with dietary polyphenol-rich grapes was shown to significantly increase the Lactobacillus population in the ceca of broiler chickens [4].

In the small intestine, glucose transporter 1 (GLUT1), recognized as part of solute carrier family 2 (SLC2A1), facilitates glucose transport across the apical surface of the enterocytes, whereas glucose and fructose transport across the basal side of the enterocytes and into the blood circulation is facilitated by GLUT2 [68]. In the current study, the inclusion of CCE in the broiler diet upregulated the expression of glucose nutrient transporters such as GLUT1, GLUT2, and SGLT-1, which are responsible for the transportation of fructose, galactose, mannose, and glucosamine. This can be attributed to the presence of anthocyanins in CCE that are characterized by α-glucosidase inhibitor activity and the capacity to combine with and activate peroxisome proliferator-activated receptor gamma (PPAR*γ*) [69]. The activation of PPAR*γ* accelerates lipid metabolism and glucose uptake by increasing the actions of insulin in glucose utilization in animals [70]. The enhanced expression of GLUT2 can regulate the digestion and absorption of poultry by modulating food consumption by controlling the feedback signal to the brain [71]. Additionally, cornelian cherry extract administration can initiate the uptake of glucose by tissues such as muscle and participate in increasing muscle mass in poultry [72,73]. Moreover, the upregulation of GLUT1 and GLUT2 can enhance their absorptive functions and increase the final body weight of broiler chickens via increasing nutrient transporter expression in the small intestine [74,75]. Additionally, plant-rich phenolics could promote the growth performance of broilers [41] through their potential to improve the antioxidant status of the gut [42]. Antioxidant capacity is a critical factor in poultry health that affects meat quality after slaughter. The radical scavenging ability is linked to the rich polyphenolic compound composition [76]. Animals have developed effective methods of protection against oxidative stress. SOD can eliminate superoxide anion free radicals, and GSH-PX and catalase can catalyze hydrogen peroxide decomposition [77]. Lipid peroxidation is produced by high levels of free radicals, and it leads to an increase in the content of MDA, the end product of lipid oxidation [78]. Levels of thiobarbituric acid reactive substance (MDA) are biomarkers for assessing the lipid peroxidation degree [79]. To the best of our knowledge, there are no data available on the impact of CCE supplementation on the expression of genes encoding the antioxidant enzymes in broilers. In the current study, the expression of antioxidant-related genes (SOD, GPX, and catalase) was upregulated in breast meat by increasing the CCE level. Chickens’ breast meat that was not supplemented with CCE had elevated MDA levels and, as a result, lowered oxidative stability, compared with groups supplemented with CCE. Thus, dietary supplementation with CCE had a postmortem effect of decreasing the rate of lipid oxidation by decreasing the MDA content in breast meat, even after 90 days of storage. Similarly, the total antioxidant capacity and phenolic content in the broilers’ breast meat improved following dietary supplementation with pomegranate peel extract [80]. Additionally, previous studies showed that lipid oxidation in chicken meat was reduced by feeding with dietary antioxidants, such as plant extracts rich in phenolic compounds [81,82]. A similar positive effect of dietary phenolic compounds on TBARS in breast meat was detected [83]. 

The higher antioxidant capacity of CCE may be related to the higher polyphenol and flavonoid contents [84], which augment the ability to scavenge radicals. Parallel results support the idea that lipid oxidation could be prevented by fortifying flavonoid antioxidants in animal feed [85]. Furthermore, supplementation of the broiler diet with blackcurrant-extract-rich-polyphenolic compounds enhanced the oxidative stability of their meat after 90 days of frozen storage [86].

Additionally, the reduced TBARS value was useful for lengthening the shelf time of meat products and improving the meat quality [11]. Additionally, the increased total phenolic content in the breast meat of broilers fed increased levels of CCE compared with broilers fed the control diet indicated a higher total antioxidant capacity [87], as these phenolic compounds are able to scavenger free radicals [88]. Besides this, supplementation with dietary CCE improved the DPPH scavenging activity of breast meat, especially at higher levels, even after 90 days of storage. The higher scavenging activity of DPPH indicated an increased antioxidant content in broiler meat, which has the potential to provide one proton to produce a stable DPPH2 compound, thus scavenging the free radicals [89]. Additionally, Cerit et al. [90] reported that cornelian cherry fruits have greater DPPH radical scavenging and ferric-reducing activity.

Moreover, the highest FRAP values were measured in meat enriched with higher levels of supplemental CCE. Faiz et al. [91] showed decreased TBARS values and better activity against 2,2-diphenyl-1-picrylhydrazyl (DPPH) free radicals, associated with a dose-dependent increase in the total phenolic compound concentration detected in chickens’ meat after they received different levels of citrus waste. Furthermore, Jang et al. [92] stated that the oxidative stability of the breast meat of chickens fed a diet enriched with Coptis chinensis extract was mainly attributed to the higher concentration of polyphenolic compounds. The antioxidant content present in broiler meat tended to convert ferric ion (Fe^3+^) to ferrous ion (Fe^2+^) by providing one electron. Higher metal-chelating potential after supplementation with CCE protected tissues from damage resulting from oxidation. Similarly, a higher metal ion reducing capacity was observed in meat enriched with natural antioxidants in contrast with a control treatment group [93]. Similarly, cornelian cherry extract can reduce 20.41 μmol of Fe^2+^/g of solution [13].

## 5. Conclusions

Supplementation with cornelian cherry extract, which is rich in antioxidants, improved the growth performance of broiler chickens via several mechanisms, such as increasing the favorable probiotic populations and lowering the concentration of harmful bacteria, such as *E. coli* spp. The modulation of genes expression responsible for glucose absorption and antioxidant enzymes indicates that CCE can play an effective role in the previously mentioned molecular mechanisms. CCE can scavenge free radicals, thereby improving the antioxidant capacity and lipid peroxidation of poultry meat without affecting its chemical composition. The results demonstrate that the application of dietary CCE (200 mg/kg) is recommended in chickens’ diets to boost their growth performance, health, and meat shelf stability during long periods of frozen storage.

## Figures and Tables

**Table 1:** Ingredients and chemical composition of the basal diet (as dry matter).

Ingredients, g/kg	Starter	Grower–Finisher
Yellow corn grain	57.40	60.1
Soybean meal, 47.5%	34.66	29.00
Corn gluten, 60%	3.00	4.00
Soybean oil	1.10	3.00
Calcium carbonate	1.00	1.00
Dicalcium phosphate	1.80	1.90
Common salt	0.30	0.30
Premix *	0.30	0.30
DL- Methionine, 98%	0.18	0.14
Lysine, HCl, 78%	0.16	0.16
Anti-mycotoxin	0.10	0.10
Analyzed Chemical Composition		
ME, Kcal/Kg **	3004	3158
CP %	23.01	21.10
EE %	3.63	5.55
CF %	2.66	2.53
Ca %	0.97	0.98
Available P %	0.47	0.47
Lysine %	1.37	1.22
Methionine %	0.56	0.51

* Supplied per kg of diet: Vitamin A, 12 000 IU; Vitamin D3, 2200 IU; Vitamin E, 26 IU; Vitamin K3, 6.25 mg; Vitamin B1, 3.75 mg; Vitamin B2, 6.6 mg; Vitamin B6, 1.5 g; Pantothenic acid, 18.8 mg; Vitamin B12, 0.31 mg; Niacin, 30 mg; Folic acid, 1.25 mg; Biotin, 0.6 mg; Fe, 50 mg; Mn, 60 mg; Cu, 6 mg; I, 1 mg; Co, 1 mg; Se, 0.20 mg; Zn, 50 mg; Choline chloride, 500 mg; ** ME calculated according to National Research Council 1994; ** ME, metabolic energy; CP, crude protein; EE, ether extract; CF, crude fiber; Ca, calcium; P, Phosphorus.

**Table 2:** Primer sequences and target genes used for Q-PCR reactions.

Gene	Gene Full Name	Primer Sequence (5′–3′)	Reference No
Glucose transporters			
*GLUT1*	Glucose transporter 1	F-TCCTCCTGATCAACCGCAAT R-TGTGCCCCGGAGCTTCT	NM_205209.1
*GLUT2*	Glucose transporter 2	F-TGATCGTGGCACTGATGGTT R-CCACCAGGAAGAC↓GGAGATA	NM_207178.1
*SGLT-1*	Sodium-dependent glucose transporter	F-TGCCGGAGTATCTGAGGAAG R-CCCCATGGCCAACTGTATAA	XM_015275173.2
Antioxidant related genes			
*GPX1*	Glutathione peroxidase	F- GCTGTTCGCCTTCCTGAGAG R- GTTCCAGGAGACGTCGTTGC	NM_001277853.1
*SOD1*	Superoxide dismutase	F- CACTGCATCATTGGCCGTACCA R- GCTTGCACACGGAAGAGCAAGT	NM_205064.1
*CAT*	Catalase	F- TGGCGGTAGGAGTCTGGTCT R- GTCCCGTCCGTCAGCCATTT	NM_001031215.1
House keeping			
*GAPDH*	Glyceraldahyde-3-phosphate dehydrogenase	F-GGTGGTGCTAAGCGTGTTA R-CCCTCCACAATGCCAA	NM205518
*TBP*	TATA-binding protein	F: GTCCACGGTGAATCTTGGTT R: GCGCAGTAGTACGTGGTTCTC	Acc:8484

**Table 3:** Effect of different levels of cornelian cherry extract (CCE) on growth performance and nutrient digestibility of broiler chickens.

CCE (mg/kg Diet)							
Parameters	0	50	100	200	400	*p*-Value	SEM
Total growing period							
BW (g/bird)	2423 ^c^	2439 ^b,c^	2508 ^b^	2649 ^a^	2503 ^b^	<0.001	14.10
BWG (g/bird)	2377 ^c^	2393 ^b,c^	2462 ^b^	2603 ^a^	2457 ^b^	<0.001	14.12
FI (g/bird)	4331 ^b^	4219 ^c^	4224 ^c^	4503 ^a^	4400 ^b^	<0.001	17.53
FCR	1.82 ^a^	1.76 ^a,b,c^	1.72 ^c^	1.73 ^b,c^	1.79 ^a,b^	<0.001	0.01
Digestibility %							
Dry matter	69.37 ^d^	71.87 ^b,c^	73.33 ^a,b^	74.80 ^a^	71.54 ^c^	<0.05	0.50
Crude protein	62.45 ^c^	63.63 ^b,c^	65.92 ^a,b^	69.22 ^a^	63.69 ^b^	<0.001	0.40
Crude fiber	27.30	27.37	27.95	29.47	29.18	<0.20	0.60

BW (body weight); BWG = body weight gain; FI = feed intake; FCR = feed conversion ratio; Number of birds/replicates = 10; ^a–d^ Means within a row carrying different superscript letters denote significant differences (*p* < 0.05).

**Table 4:** Effect of different levels of cornelian cherry extract (CCE) on serum biochemical parameters of broiler chickens.

	CCE (mg/kg Diet)						
Parameter	0	50	100	200	400	*p-*Value	SEM
ALT, U/L	20.82	20.78	20.98	20.60	19.58	0.18	0.40
AST, U/L	55.16	55.40	54.00	54.64	53.58	0.06	0.48
Uric acid, mg/dL	5.70	5.96	5.74	6.04	5.91	0.55	0.06
Creatinine, mg/dL	0.96	1.02	0.98	1.1	0.94	0.69	0.01
Total Cholesterol, mg/dL	109.16 ^a^	109.30 ^a^	107.76 ^a^	109.34 ^a^	100.78 ^b^	<0.001	3.62
TGs, mg/dL	61.18	60.78	61.24	60.32	60.28	0.34	0.38
HDL-C, mg/dL	43.34	44.58	44.48	44.78	43.90	0.17	0.36
LDL-C, mg/dL	53.58 ^a^	52.56 ^a^	51.03 ^a^	52.49 ^a^	44.82 ^b^	0.02	4.20
VLDL-C, mg/dL	12.23	12.16	12.24	12.06	12.00	0.34	0.02
Total protein (g/dL)	4.43	4.40	4.41	4.47	4.44	0.96	0.03
Albumin (g/dL)	2.28	2.25	2.32	2.25	2.25	0.97	0.03
Globulin (g/dL)	2.15	2.16	2.09	2.21	2.19	0.96	0.05

^a,b^ Means with different superscripts within the same row differ significantly (*p* < 0.05); ALT: Alanine aminotransferase, AST: Aspartate aminotransferase, TGs: Triglycerides, HDL-C: high- density lipoprotein cholesterol, LDL-C: low-density lipoprotein cholesterol, VLDL-C: very-low density lipoprotein cholesterol.

**Table 5:** Effect of different levels of cornelian cherry extract (CCE) on breast and thigh muscle chemical analysis of broiler chickens % on wet basis.

CCE (mg/kg Diet)							
Parameters	0	50	100	200	400	*p*-Value	SEM
Breast Muscle Analysis % of Wet Basis							
DM	25.76	25.26	25.61	24.73	25.35	0.614	0.21
CP	23.61	23.04	24.19	23.24	23.92	0.322	0.19
EE	4.52	4.52	4.63	4.64	4.56	0.949	0.06
Ash	1.28	1.20	1.27	1.22	1.25	0.864	0.02
Thigh Muscles Analysis% of Wet Basis							
DM	28.42	28.34	27.21	26.52	28.36	0.273	0.34
CP	22.06	20.66	20.38	21.95	21.74	0.051	0.24
EE	6.94	7.21	6.62	6.31	6.91	0.087	0.11
Ash	1.18	1.23	1.29	1.11	1.24	0.399	0.03

Dry matter: DM, crude protein: CP, ether extract: EE.

**Table 6:** Effect of different levels of *Cornelian cherry extract* (CCE) on cecal microorganisms (Log_10_ cfu/g fresh digesta) of broiler chickens at slaughter.

	CCE (mg/kg Diet)						
	0	50	100	200	400	*p*-Value	SEM
*Bifidobacterium*	6.17 ^b^	6.57 ^b^	7.70 ^a^	7.90 ^a^	8.17 ^a^	<0.01	0.20
*Lactobacillus*	6.50 ^d^	6.70 ^d^	7.73 ^c^	8.13 ^b^	8.87 ^a^	<0.001	0.10
*Escherichia coli*	8.23 ^a^	7.9 ^ab^	7.23 ^b^	6.47 ^c^	6.27 ^c^	<0.008	0.16

Number of birds/replicate = 10. ^a–d^ Means within a row carrying different superscript letters denote significant differences (*p* < 0.05).

**Table 7:** Effect of different levels of cornelian cherry extract (CCE) on breast meat total phenolic content oxidative stability and lipid peroxidation during freezing storage at −20 °C.

CCE (mg/kg Diet)							
Parameters	0	50	100	200	400	*p*-Value	SEM
TPC at d 7 of storage	68.71 ^e^	111.06 ^d^	124.06 ^c^	131.68 ^b^	143.72 ^a^	4.91	<0.001
TPC at d 90 of storage	49.77 ^d^	96.71 ^c^	109.51 ^b^	121.95 ^a^	128.02 ^a^	3.27	<0.001
DPPH assay at d 7 of storage	86.99 ^e^	118.20 ^d^	133.67 ^c^	148.58 ^b^	155.88 ^a^	4.45	<0.001
DPPH at d 90 of storage	77.58 ^e^	106.93 ^d^	124.61 ^c^	134.58 ^b^	144.03 ^a^	3.89	<0.001
FRAP assay at d 7 of storage	229.44 ^e^	435.81 ^d^	447.99 ^c^	514.62 ^b^	618.37 ^a^	8.09	<0.001
FRAP assay at d 90 of storage	175.44 ^d^	292.34 ^c^	301.89 ^c^	369.88 ^b^	474.55 ^a^	7.51	<0.001
MDA content at d 7 of storage	0.47 ^a^	0.26 ^b^	0.22 ^c^	0.15 ^d^	0.19 ^d^	0.12	<0.03
MDA content at d 90 of storage	0.67 ^a^	0.46 ^a^	0.30 ^b^	0.28 ^b^	0.21 ^c^	0.03	<0.008

TPC = Total phenolic contents; DPPH = 2,2-Diphenyl-1-picrihydrzyl; FRAP = Ferric reducing antioxidant power; MDA = malondialdehyde; Number of birds/replicate = 10; ^a–e^ Means within a row carrying different superscript letters denote significant differences (*p* < 0.05).

## Data Availability

The data source was sent to a section of nonpublished material.
